# Primary Squamous Cell Carcinoma of the Uterus and Not the Cervix—A Case Report and Literature Review

**DOI:** 10.1155/crog/7140494

**Published:** 2025-06-13

**Authors:** Judy Hayek, Mariem Del Rio, Yongmei Yin, Margaux J. Kanis

**Affiliations:** ^1^Department of Obstetrics and Gynecology, Maimonides Medical Center, Brooklyn, New York, USA; ^2^Department of Obstetrics and Gynecology, NewYork-Presbyterian (NYP) Brooklyn Methodist Hospital, Brooklyn, New York, USA; ^3^Department of Pathology, NewYork-Presbyterian (NYP) Brooklyn Methodist Hospital, Brooklyn, New York, USA; ^4^Division of Gynecologic Oncology, NewYork-Presbyterian (NYP) Brooklyn Methodist Hospital, Brooklyn, New York, USA

**Keywords:** adjuvant radiation, endometrial, HIV, primary surgery, squamous cell

## Abstract

Primary endometrial squamous cell carcinoma (PESCC) is a rare pathology. Data regarding diagnosis and treatment is limited and is solely based on case reports and series. We report a unique case of a postmenopausal woman with a history of HIV and anal cancer s/p radiation therapy 8 years prior. The patient's presenting diagnosis was malodorous vaginal discharge refractory to antibiotics. The patient had a significant delay in diagnosis since the onset of symptoms despite extensive workup but eventually underwent surgical staging by gynecologic oncology with a diagnosis of Stage IB PESCC. Despite her reported history of anal cancer for which she received an incomplete course of pelvic radiation, the case was discussed at tumor board and the patient was deemed a candidate for external beam radiation therapy. In our report, we discuss, in detail, the patient's presentation, course, and treatment and review the literature.

## 1. Introduction

Endometrial cancer is the fourth most common cancer among women in the United States but the most frequently diagnosed gynecologic malignancy [1]. The American Cancer Society estimates 66,200 new cases of cancer of the uterus and 13,030 cancer-related deaths in 2023 [1]. Squamous cell carcinoma remains a rare pathological entity mainly described through case series and reports. We present a unique case of primary squamous cell carcinoma of the uterus in a patient with HIV and a remote history of anal cancer s/p radiation therapy.

## 2. Case Report

**Figure 1:**
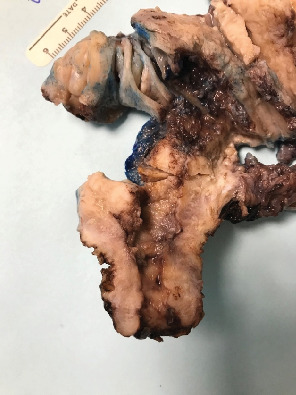
Gross specimen. Tumor size: 5.0 × 3.5 × 2.2 cm. Involves entire endometrium, extended from fundus to lower uterine segment. Negative cervix.

**Figure 2:**
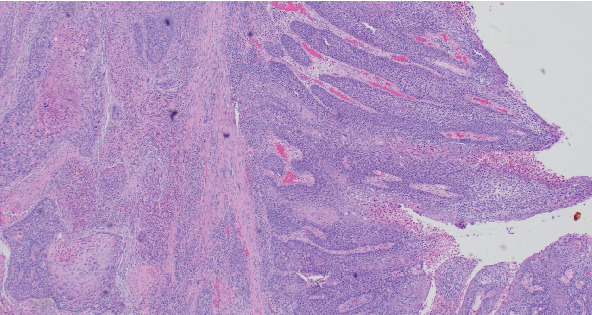
Tumor shows in situ and invasive component (HE, 10 × 10).

**Figure 3:**
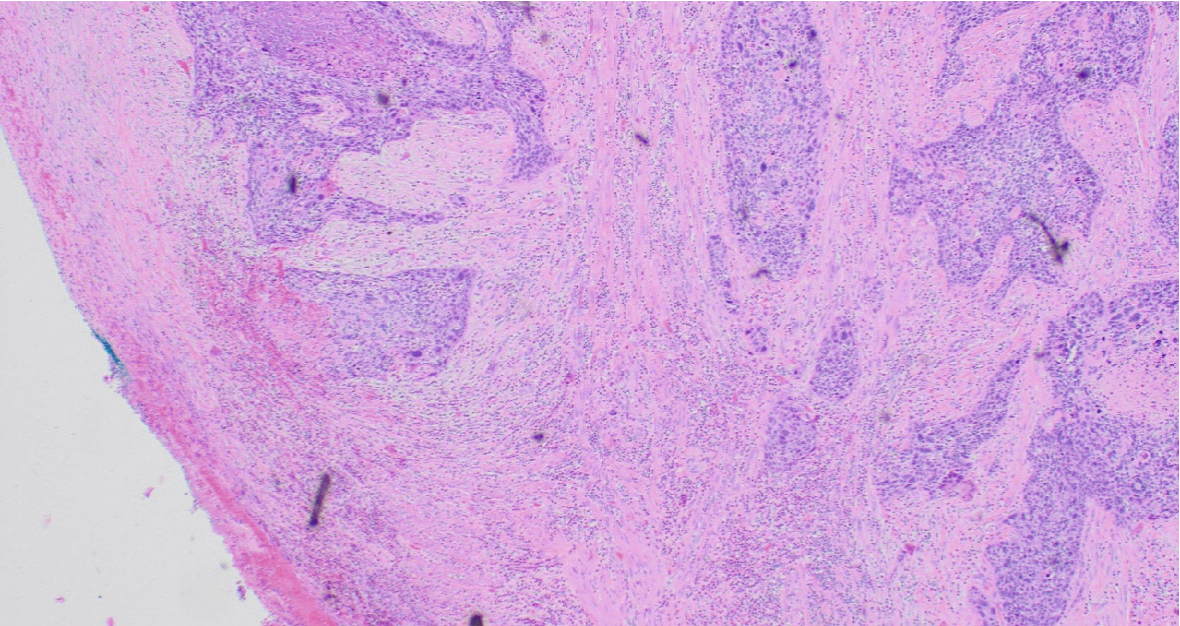
Myometrial invasion: > 90% (HE, 4 × 10).

**Figure 4:**
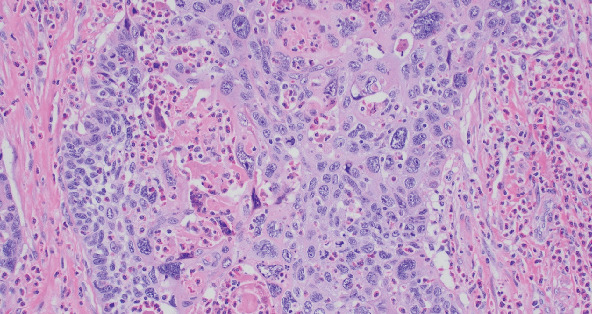
Squamous cell carcinoma, not otherwise specified, moderately differentiated (HE, 20 × 10).

A written informed consent was obtained from the patient. Our patient is a 59-year-old gravida 4 para 3 postmenopausal female with a past medical history of HIV and anal cancer. She was diagnosed with anal cancer 8 years prior to her current presentation and underwent chemoradiation therapy. The patient was referred to gynecologic oncology by her primary provider after a Papanicolaou smear revealed ASCUS and human papillomavirus (HPV) positive cotesting (negative for HPV 16 and 18) in September 2022. She reported a history of cervical dysplasia (HSIL encompassing CIN 2/3 status post-LEEP) in 2020 and a 1-year history of abundant purulent vaginal discharge refractory to multiple courses of antibiotics. A colposcopy was performed in October 2022; cervical biopsies were negative for malignancy while an endometrial biopsy revealed at least squamous cell carcinoma in situ; invasion could not be excluded, but no endometrial cells were seen. A pelvic ultrasound revealed a thickened and irregular endometrial lining measuring up to 32 mm. The patient was then taken for a hysteroscopy, dilation and curettage, and cervical biopsy in November 2022. Findings included an 8 cm anteverted uterus cervix that was flushed with the vagina, and multiple polypoid masses were seen in the uterine cavity on hysteroscopy. The cervical biopsy revealed metaplastic squamous mucosa of the cervix with no dysplasia; endocervical curettage revealed benign squamous epithelium with focal condylomatous change. The endometrial curettage failed to obtain endometrial tissue, but high-grade squamous intraepithelial lesion (CIN3) with severe dysplasia was seen. Biopsy results were discussed with the patient in detail, and given high suspicion for cancer despite inconclusive workup, she was counseled on definitive surgery for which she consented. She underwent a total robotic hysterectomy with bilateral salpingo-oophorectomy, pelvic lymphadenectomy, and cystoscopy with ureteral stent insertion in December 2022. Extensive pelvic adhesions were seen, and ureteral stenting was performed for better identification of the ureters to avoid any injuries (Figure 1). The final pathology was consistent with endometrial squamous cell carcinoma, FIGO Stage IB, with 96% myometrial invasion (Figures 2, 3, and 4). No cervical stroma invasion was identified. Vascular invasion was noted with one vessel involvement in the lower uterine segment, but no lymphatic or regional lymph node involvement was identified. All margins were negative for invasive carcinoma. Immunopathology studies revealed positive mutant p53, p16, p63, 40, a high Ki67, focally positive Pax-8, PDL-1 positive, MMR proficient status, and HER-2 negative. A chest computed tomography scan was performed to complete staging, with no evidence of metastatic disease noted. Molecular tumor profiling was performed and resulted in PDL-1 positive, MMR proficient, and PIK3CA mutation. The case was then discussed among the gynecologic and radiation oncologists during tumor board; the consensus was that external beam radiation therapy would be the recommended approach for adjuvant treatment. Upon follow-up with radiation oncology, a discussion was had with the patient regarding high-risk features of her tumor including p53 mutation and 96% myometrial invasion, and she was deemed a candidate for pelvic radiation at that time. She was, however, lost to follow-up until March 2023. The patient completed external beam radiotherapy to the pelvis 5040 cGy in 28 fractions followed by high dose rate brachytherapy boost of 600 cGy in two fractions in April 2023. She has been without evidence of disease for 19 months after the completion of adjuvant RT and 23 months since her surgical staging.

## 3. Discussion

Primary squamous cell carcinoma of the uterus is a rare subentity of uterine carcinoma that has been only described in the literature through case reports. The criteria for diagnosis were established in 1928 by Fluhmann and included exclusion of cervical carcinoma extending into the endometrium, coexistent endometrial adenocarcinoma, and contiguity between the endometrial cancer and the squamous cervical epithelium [2].

The etiology of squamous cell carcinoma of the uterus is unknown given its rarity. Goodman et al. found 60 cases in a review of 1182 cases of uterine cancer at Massachusetts General Hospital between 1975 and 1993. They reported that the average age of diagnosis was 67 years, and the most common presenting symptom was vaginal bleeding. Other symptoms, including vaginal discharge, pelvic pain, and uterine mass, were encountered. Risk factors for primary endometrial squamous cell carcinoma included chronic pyometra, pelvic radiation, and nulliparity [3]. More recently, Bogani et al. described a series of four cases in which all patients were > 65 years of age. In two of these cases, risk factors included pelvic inflammatory disease and cervical stenosis [4]. Similarly, our patient presented with vaginal discharge and pyometra and had a history of pelvic radiation in the past.

Multiple hypotheses have been suggested to explain the pathogenesis of primary endometrial squamous cell carcinoma. Horn and Bilek, in 1993, suggested that PESCC is the result of a bidirectional differentiation of pluripotent endometrial precursor cells [5]. In 1995, Yamamoto et al. observed that this malignancy may arise from heterotopic cervical tissue [6]. More recent publications investigate the role of HPV infection [7]. However, the latter theory has proven to be controversial as HPV has not been detected in most cases reported. Some more convincing hypotheses describe ichthyosis uteri, the squamous differentiation of the endometrium, as a result of chronic uterine irritation. These irritants may be the result of radiation, a chronic inflammatory process such as pelvic inflammatory disease or cervical stenosis, or simply age [4, 8].

In the era of molecular analysis, attempts have been made to challenge the early hypotheses and better define the criteria for the diagnosis of PESCC. In our literature review, we found two studies describing the molecular characteristics of PESCC [9, 10]. Giordano et al. found an aberrant expression of P53, while the expression of p16 and PTEN was absent [9]. Similarly, Hopkins et al. identified five cases of PESCC for which molecular analyses and next-generation sequencing were performed. All five cases demonstrated an aberrant P53 pattern and were negative for p16, estrogen receptor, and progesterone receptor. Moreover, next-generation sequencing revealed concurrent P53 and CDKN2A mutations in 4/5 cases. Mutations in mismatch repair genes, POLE, RAS, EGFR, ARID1A, C-myc, BSL2, MET, BRAF, and ERBB2, were not identified [10]. The molecular profile described by Hopkins et al. resembles that of high-grade endometrioid and serous adenocarcinomas of the uterus and could potentially explain the aggressive nature of endometrial squamous cell carcinoma. In fact, most cases described in the literature reiterate the overall poor prognosis of PESCC [3, 11].

Given the scarcity of the data available, there are no definitive recommendations for treatment and plans should be individualized [12]. Many cases reported support the utility of surgery followed by radiation therapy [13, 14]. While the radicality of the surgery depends on the diagnostic information gathered prior, establishing a diagnosis prior to definitive surgery can be quite challenging. Of the 60 cases of primary endometrial squamous carcinoma reviewed by Goodman et al., only 50% of women had a preoperative diagnosis despite a thorough workup. Furthermore, in their large review, they noted an average treatment delay of 11.5 months. Fifty-eight patients of those reviewed underwent total abdominal hysterectomy with bilateral salpingo-oophorectomy as primary treatment. Eighty percent of Stage I disease survived with a median follow-up of 32 months. The survival rate for Stage III tumors was 20% and nearly null for Stage IV [3].

In a case report by Caulkins et al., they described a concurrent case of Stage IB primary squamous endometrial squamous cell carcinoma and Stage IA fallopian tube cancer. The patient was treated with six cycles of cisplatin and paclitaxel without radiation. She was reported to be in remission for at least 2 years from treatment [15]. There has not been any consistency in treatment modalities in the published literature; however, given the aggressive nature of the disease, chemotherapy may be of value; its utility in the up-front setting for early-stage disease remains a question yet to be answered. We tend to follow the treatment guidelines for early adenocarcinoma of the uterus; however, upon further investigation of the molecular profile of the tumor, chemotherapy and immunotherapy may have a role in the future.

In conclusion, we describe a rare case of a menopausal patient with HIV and a history of anal cancer for which she received pelvic radiation therapy, who was diagnosed with primary squamous cell cancer of the uterus. This case particularly demonstrates a challenge given the patient's initial delay in diagnosis and her vague history of pelvic radiation therapy. More studies are needed for clearer guidance on treatment for such an aggressive uterine pathology.

## Data Availability

The data that support the findings of this study are available from the corresponding author upon reasonable request.
